# Barriers and facilitators to deliberate practice using take-home laparoscopic simulators

**DOI:** 10.1007/s00464-018-6599-9

**Published:** 2018-11-19

**Authors:** Vivienne I. Blackhall, Jennifer Cleland, Philip Wilson, Susan J. Moug, Kenneth G. Walker

**Affiliations:** 1 Scottish Surgical Simulation Collaborative, Royal College of Surgeons of Edinburgh and Royal College of Physicians and Surgeons of Glasgow, Edinburgh, UK; 2 Highland Academic Surgical Unit, Raigmore Hospital and Centre for Health Science, Inverness, UK; 3 Centre for Health Education Research and Innovation, University of Aberdeen, Aberdeen, UK; 4 Centre for Rural Health, University of Aberdeen, Aberdeen, UK; 5 Department of General Surgery, Royal Alexandra Hospital, Paisley, UK

**Keywords:** Deliberate practice, Simulation, Surgical training, Laparoscopy

## Abstract

**Background:**

Several regions in the UK and Ireland have delivered home-based laparoscopic simulation programmes in an attempt to progress surgical trainees’ skills through deliberate practice. However, engagement with these programmes has been poor. This study aims to uncover the barriers to engagement with home-based simulation, with a view to developing an improved programme.

**Methods:**

This was a qualitative study using focus groups with key stakeholders including junior surgical trainees, consultants/attendings and simulation faculty. Data were collected across four regions in three countries. Data were audio-recorded, transcribed and a thematic analysis was performed using NVivo software.

**Results:**

Sixty-three individuals were interviewed in 12 focus groups (43 trainees, 20 trainers). Trainees cited competing commitments as a barrier to engaging with home-based simulation. They tended to focus on scoring ‘points’ towards career progression rather than viewing tasks as interesting, or associated with personal development. Their view was that this approach is perpetuated by the training system, which rewards trainees for publications and exams, but not for operative skill. Trainees were unsatisfied with metric feedback and wanted individual feedback from consultants (attendings). Trainees perceived consultants as lacking interest in the programmes and training in general. However, some consultants were unaware of the programmes being delivered and others felt lacking in confidence to deliver the necessary training.

**Conclusions:**

Scheduled simulation sessions which provide trainees with the opportunity for consultant feedback may improve engagement. Tackling the ‘point-scoring’ culture is more challenging. This could be addressed by modified assessment structures, greater recognition and accountability for trainers, and recognition and funding of simulation strategies including in-house skills sessions.

The delivery of surgical training has changed significantly over the past few decades. There has been a shift away from the traditional apprenticeship model towards outcomes and competency-based educational frameworks, and a reduction in the total number of hours available for training resulting in less time for “on-the-job” learning [1]. These changes, coupled with an increased emphasis on patient safety, have necessitated the development of alternative teaching and learning approaches, including simulation [2]. Evidence suggests that simulation-based education (SBE) can have an important role in the development of surgical technical skills and confidence, as well as decreasing reliance on training using real patients and improving clinical and operating room time efficiency [3–7].

McGaghie et al. describe the opportunity for deliberate practice (DP), and the integration of DP into the curriculum, as essential for effective simulation-based education (SBE) [8]. Deliberate practice is the intentional repetition of a skill (either technical or cognitive) with the provision of feedback to correct errors and improve performance [9]. In surgical training, DP using portable laparoscopic box simulators is associated with the acquisition of laparoscopic skills which may, in turn, be transferred to the operating theatre [10–12]. However, there is some evidence that trainees tend not to avail themselves of the opportunity to engage with practice [13].

This was our experience in our own attempt to incentivise deliberate practice on take-home simulators by trainees in the two Scottish Core Surgical training programmes. The focus of our programme during the Incentivised Laparoscopy Practice Study (ILPS) [14] was to provide incentives for core surgical trainees to perform DP using take-home simulators and then to assess the resulting trainee engagement with the programme and to measure their laparoscopic motor skills. Trainees were given a portable laparoscopic simulator, an online module to practice, metric and personalised objective feedback, and an incentive in the form of an eCertificate. The online module comprised six tasks, appropriately challenging for our beginner cohort of trainees, demonstrating both construct and content validity [15]. The eCertificate was awarded to participants on attaining certain metric scores and producing a videoed performance to a certain standard [16]. The eCertificate would then cue trainers to allow the trainee access to ‘first operator’ tasks in the live theatre. ILPS was not mandatory but was offered to core surgical trainees as an adjunct to formal training.

Although performances improved in some participants, the study found generally poor engagement by trainees with ILPS. Anecdotal evidence from elsewhere in the UK suggests that poor engagement with non-mandatory training adjuncts is not unique to our programme, but is widespread. However, we currently have little understanding of the barriers to engaging with deliberate practice on take-home simulators [9].

A systematic review by Gostlow et al. reflected upon the barriers and motivators to participation in simulated laparoscopic skills and found that lack of available time was the greatest factor influencing engagement with practice [17]. However, the focus of Gostlow’s review was ‘in-house’ rather than home-based simulation and trainees were of a variety of training grades. Barriers and motivators to engagement were examined as secondary outcomes through surveys (with the primary outcome typically representing the amount of time spent training). The authors indicated that further large scale research was required to evaluate barriers and motivators to engagement with simulation [17].

In short, while there is much evidence that DP accelerates the acquisition of expert performance, if trainees do not engage with DP, then they may struggle to gain necessary skills within limited training hours and restricted access to patients. If SBE and DP are to be integrated effectively into surgical training, we need to identify barriers and facilitators to trainee engagement [9].

Thus, our aim was to explore key stakeholder views of the barriers and facilitators related to engagement with a take-home deliberate practice simulation programme for laparoscopic skills.

## Materials and methods

This was a qualitative study using focus groups for data collection. This approach was chosen because focus groups provide insight into complex behaviour and motivation [18] and have been used previously to establish reasons for poor take-up of services [19].

In the Scottish programme, the views of the following ILPS stakeholders were sought:

- Two groups of core surgical trainees: one ‘familiar’ group involved with the original ILPS study [14], the other a naïve group (in terms of the original study) but familiar with the simulation equipment.
- Core Surgery Training programme directors.
- Consultant surgeons who train core surgical trainees and use laparoscopic surgery.
- ILPS faculty, including the designers of the laparoscopic simulator equipment.

To maximise the transferability of the data, we also sought the views of trainees and faculty members from three other regions where programmes similar to ILPS had been implemented (two English regions, one Irish). Although the four programmes were similar, there were also some differences (Table 1). For example, the English regions mandated their programme and the Irish programme did not include any form of metric feedback data.

**Table 1:** Key features of each of the four training programmes

Feature of programme	Scotland	Ireland	Wessex	Bristol
Access to portable simulator	✓	✓	✓	✓
Simulator type	EoSim	EoSim	EoSim	EoSim
Access to online training module	✓	✓	✓	✓
Module type	EoSim core	EoSim core	EoSim core	EoSim core
Technical support	✓	✓	✓	✓
Clear goals	✓	✗	✓	✓
Metric feedback	✓	✗	✓	✓
Individualised feedback from superior	✓	✗	✓	✓
Attainment of eCertificate	✓	✗	✓	✓
Mandated	✗	✗	✓	✓

All trainees were given an EoSim simulator (EO Surgical, Edinburgh) with access to the online core training module (including tasks such as peg threading, precision cutting and dice stacking). In keeping with the principles of deliberate practice, all trainees had access to a portable simulator, giving them an opportunity to engage with repetitive practice. In addition, clear, achievable goals were set and most groups of trainees were provided with at least one form of feedback

Recruitment of core trainees, trainers, faculty and training programme directors (TPDs) was conducted via emails from the Scottish Surgical Simulation Collaborative (SSSC). Of 122 invitations sent, 63 positive responses were received (51.6%) (Trainees 43/80; Trainers: 6/24; faculty 12/16; TPDs: 2/2). Positive responses were followed up by emails providing more information about the study. All focus groups were facilitated by VB, who received training in this respect from JC who is an experienced qualitative researcher. Different groups of participants (i.e., trainees, trainers) were interviewed separately in order to encourage open discussions. Only the researcher and the participants were present during the focus groups. Participants were aware that the lead researcher was conducting the study as part of a higher research degree.

We identified a number of surgical training events during the time period of the project where we were able to interview participants in groups. This included the Association of Surgeons of Great Britain and Ireland (ASGBI) conference, Scottish Surgical Bootcamp, Faculty of Surgical Trainers Annual Meeting and the Irish Surgical Bootcamp.

A focus group question guide was developed on the basis of the literature and informal feedback from the ILPS faculty and participants (Appendix 1). While the questions were slightly different for those who had knowledge of the ILPS or equivalent programmes and naïve trainers/trainees, they included: exploring participants’ understanding of deliberate practice and its role in surgical training; barriers and facilitators related to uptake; potential means to overcome barriers.

Quotations from individuals were identified using a unique code specific to their original vocational group, followed by a number:

- Familiar trainees: FT
- Naïve trainees: NT
- Consultant trainers: CO
- Training programme directors: TPD
- Faculty: FC

Ethical approval was sought and granted from the University of Aberdeen College Ethics Review Board (Reference: CERB/2017/3/1430). All participants provided written consent before taking part in a focus group.

Interviews were audio recorded with participant permission. The recordings were transcribed in full for analysis by an independent, professional transcription service. NVivo software was used to store, manage, and analyse the transcripts. Initial data coding and analysis of the transcribed interviews were inductive, using thematic analysis to generate a coding scheme which was then used to code all data. This was performed in accordance with Braun and Clark’s six-point framework for thematic analysis [20]. Analysis progressed via regular team meetings and telephone/Skype discussions, where coding was refined and comparisons were explored. Comparisons were made between codes and participants to explore differences and similarities in participants’ perspectives.

## Results

A total of twelve focus groups were undertaken with 63 individuals (two training directors (TPDs), twelve faculty, six consultant surgeons and 43 trainees). Four main themes were identified in the data: trainee motivation, provision of performance feedback, trainer involvement, and the influence of surgical systems. These are discussed in more detail below.

### Trainee motivation

Core surgical trainees reported multiple, competing demands upon their time which impacted upon their ability to engage with home-based simulation.

TPD1: “trainees are very busy, they have a lot of requirements that they have to fulfil”

FT229: “I had to deal with [home based simulation] on top of going to work, doing audits, doing research, doing presentations”

The data suggest that trainees are motivated toward undertaking tasks which are explicitly and directly associated with career progression, rather than pursuits which they find interesting or are associated with personal development. They prioritise tasks which score points at interview or contribute towards their end-of-year assessment.

NT10: “I will look at the list of courses that gets scored on your CV, and tick those off, rather than go to a conference that I am interested in”.

FT27: “[I prioritise] Exams, WBAs [work based assessments], research and projects, getting points interviews for ST3 really”

Unfortunately, this can be to the detriment of their technical skills.

NT7: “having good dexterity becomes less of a priority, because you spend time going to courses, case reports, things that will get you points”

Whilst trainees are focused on doing what is necessary to score points and secure a job:

NT13: “anything for career progression. Tick boxes, if it gets us ahead on schemes”

Consultant trainers expect their trainees to devote their time to the study of surgery and becoming good operators:

TPD2: “you need to know that they have observed, they have gone away and practiced and they know how to put ports in or actually open a mid-line incision”.

These contrasting perspectives on surgical training result in trainers perceiving trainees as failing to take responsibility for their own personal development. Yet, the data suggest that trainee attitudes are related, at least to some extent, to the culture and structure of surgical training: the structure of current surgical training schemes appears to support a ‘point scoring’ approach, rather than rewarding trainees for ‘becoming good operators’.

NT14: “the system promotes tick boxes, rather than actually being a better surgeon. You have to play the game”.

The data suggest that some trainees might have been less than honest in order to avoid participation in the programmes, falsely claiming that they encountered technical issues:

FC6: “we’ve come across the issue where trainees say they’ve had technical difficulties but they actually haven’t. I can go back and check whether or not they [have]”

Supporting the implication that core surgical trainees prioritise point scoring over personal development is the finding that mandating the programme is associated with increased module completion:

FT8: “we were told: there’s six modules to complete, this is the standard, once you get it, you got your certificate. At the ARCP [Annual Review of Competency Progression], if you’d done it, you were fine”.

Mandating simulation is not however associated with a sustained commitment to practice: it seems instead to reinforce the ‘tick box’ culture rather than challenging it.

FT9: “once I’d completed it, I’ve got my certificate, I haven’t gone back to it”.

This may be because trainees fail to see the validity and clinical correlation of the tasks themselves:

NT5: “but in what scenario do we actually stack cubes in a person’s abdomen, how could I apply that skill to real life?”

Trainees did not acknowledge that the tasks were developing transferable skills such as dexterity, and instead wanted simulated tasks which represented actual operations.

FT26: “it’s better if it’s actual models and lap[aroscopic] suturing”.

Trainees also preferred practicing on live patients:

NT3: “more to learn if you’re in theatre, you’re getting parts of operations. You’re paying more attention… it’s like high risk”.

FT 28: “I could go into theatre and just do this in theatre and practise there”.

### Provision of performance feedback

Most trainees were given metric feedback on their performance, for example, time to complete the task, hand dominance and path distance of their instruments. The time to complete the task was used as a performance marker. However, trainees were unconvinced of the trustworthiness of the metric data provided:

NT4: “I don’t think it’s able to reliably critique your performance”.

Faculty members also had concerns about the validity of the metric data and whether being able to perform a task faster translated to better performance in real life:

FC1: “they’re inaccurate. And proving that they are competent? I don’t think so”.

One region was so concerned with regard to the validity of the metric data that they did not include it in the simulation programme. Moreover, the data showed that most trainees wanted individualised performance feedback from their trainers, not metric data:

NT7: “I like the idea that when I am doing it, someone could actually watch me and give feedback”.

### Trainer involvement

In addition to providing personalised feedback, trainees were clear in the ways in which they wanted their trainers to be involved. The incentive of being rewarded with live operating was not realised for many of the trainees who participated in the original Scottish study (ILPS).

FT3: “It didn’t necessarily translate in terms of being able to do more operating”.

A major reason for this was a lack of trainer engagement with the programme, which appeared to relate to three domains: awareness, trainer skillset and lack of interest. Trainers were apparently lacking awareness of the programme.

TPD2: “we didn’t actually publicise it enough with the trainers in certain places”.

Some trainers lacked confidence in delivering training to their junior colleagues.

CO5: “we, as trainers, some of us feel that we’re not competent to deliver [training]”.

Other trainers seemed to be uninterested in the programme, and this was an additional barrier to engagement.

FT4: “My consultant didn’t really care that I was doing this, I was not getting any operating because I was doing this. So there was no incentive for me to keep doing these tasks”.

In addition, the operative reward may not have been realised because the training behaviours of the trainers may be difficult to challenge. Trainers tend to work at the pace of their own training model, where they will gradually increase the level of the trainee’s operative responsibility over time. One consultant stated:

FC5: “whether they are proficient at doing it to begin with may not affect their [operative] exposure because consultants expect a core trainee to be green and to [have to] take them through the basics of a procedure”.

This was re-iterated amongst senior trainees whom were involved with programme delivery:

FC6: “it felt like no matter how much training people did outside of theatre, when they got into theatre, every trainer just wanted to, you know, go back to basics”.

Consultant trainers want to see with their own eyes what the trainee is capable of, rather than relying upon attainment of a certificate.

FC1: “[the certificate] really isn’t worth the piece of paper it’s on. The only thing it proves is that they’ve actually spent some time on the machine. It doesn’t prove competency”.

Conversely, there was anecdotal evidence from one region that some trainers were entrusting trainees with live operating purely upon the basis of previous positive engagement with the box simulators:

FC7: “I’ve heard anecdotal stories of core trainees performing laparoscopic cases, having only had the lap trainer beforehand, supervised off the bat with a new trainer”.

### The influence of surgical systems

Two systems subthemes were identified: clinical (relating to the systems at work in the clinical workplace) and educational (relating to the way in which educational interventions are delivered).

### Clinical

Although trainees see their consultants as providing a gateway to operating, there were other systems factors which affected the realisation of an operative reward. For some trainees, the incentive failed to materialise because they were not working in a specialty which utilised laparoscopic surgery.

FT3: “At the time I was doing a plastic surgery job and I found it very difficult to motivate myself to do it because I didn’t have the opportunity to use it in practice”.

TPDs and trainers recognised the importance of explicitly connecting the simulation intervention to actual clinical practice:

CO1: “it depends on what they’re learning on the job. Unless they’re able to transfer that skill to what they’re learning in theatre they will not be motivated”.

Core surgical trainees tended to be drafted away from theatre to undertake ward duties, which in turn impacted upon the likelihood of fulfilling the operative reward:

CO4: “in [City x] the core trainees don’t get to operate because all they do is clerking in patients.

### Educational

The data suggested that simulation training needs to be explicitly built into the surgical system to encourage trainee engagement. Indeed, trainees stated that they wanted simulation training to be integrated into their normal working hours in the form of regular, scheduled ‘box trainer’ teaching sessions. Their view was that not only would these fora represent an opportunity for trainer feedback, but the sessions would allow trainees to compare notes with their peers and troubleshoot equipment problems with faculty:

FT11: “Sessions where you’re with your peers to do it together”.

FT12: “have a group session. Someone could be looking at us doing it, saying, you do it this way... That is useful”.

The notion of scheduled teaching sessions, involving an element of assessment, was also supported by one of the TPDs interviewed.

TPD2: “an assessment day lasting one or two days, towards the end of the core training programme. We could ask them to demonstrate what they’ve learned”.

## Discussion

To the best of our knowledge, this is the first study which looks explicitly at barriers to simulation-based education and deliberate home-based laparoscopic practice in surgical trainees.

Our data indicated that trainees prioritise those activities which are explicitly counted toward career progression while devaluing “non-essential” tasks even though they promote personal skills development. Reasons for this included a perceived disconnect between the simulation tasks and clinical practice, and no direct link between core surgical training assessments and the tasks. These findings suggest that the structure and culture of surgical training may not encourage engagement with certain learning activities—those which are useful for personal development, but not essential to progression. Mandating SBE may appear to address this barrier, but may actually only perpetuate a “tick box” culture [21, 22]. Essentially, assessment must be coupled to routine clinical practice if learners are to engage otherwise the task becomes an obligation rather than a learning opportunity. Recent evidence suggests that this pattern is not unique to SBE, but has been seen in work-based assessments (WBAs) [23].

The literature suggests that in order to be effective, deliberate practice should fulfil the following four domains [9]:

1. Use appropriately challenging tasks with proper sequencing
2. Provide the opportunity for repetitive practice
3. Provide immediate and informative feedback
4. Ensure that learners are adequately motivated to engage

In principle, the ILPS programme appeared to satisfy criteria one, two, and three, but in practice, the feedback component was perceived to be sub-optimal. Rather than the provision of metric feedback, trainees wanted individualised performance feedback from their trainers. Interestingly, this finding contrasts with the systematic review conducted by Gostlow et al. whereby supplementary educator feedback was not highly valued [17]. As mentioned, this may be due to key differences in methodologies between our paper and the papers included in the review.

Criterion four, ensuring learner motivation, was a major problem for all of the simulation programmes in this study. This paper has described some of the factors responsible for poor motivation amongst the trainees.

Previous studies have examined factors which motivate other groups of professionals to engage with deliberate practice. Self-improvement and client benefit were two key motivators for student teachers [24]. However neither of these featured as dominant motivators for our trainees. We had expected that metric scores, though inadequate as measures of skill, might at least be motivators as the scores improved with practice. However trainees and faculty disagreed, considering these scores to be too dissociated from reality. Similarly, trainees did not recognise the importance of deliberate practice for patient safety (their future clients), and even expressed a preference for “practising” on patients rather than a simulator. A lack of understanding surrounding the importance of deliberate simulated practice signifies the need for trainee education in this respect. Similarly, the trainee viewpoint that low fidelity equates to low value should also be challenged by presenting evidence that simulated practice using basic laparoscopic box-trainers does promote skill acquisition [10, 11]. Indeed, developing a common understanding between educationalists and trainees may help to ensure the delivery of a successful future intervention.

Incentives (in our case the eCertificate and the promise of progression in the operating theatre) may not represent the ‘magic bullet’ in terms of improving engagement. Several non-medical papers have posited that delivering well-meaning incentives may actually make engagement worse [25] because external incentives may undermine an individual’s intrinsic motivation [26].

Our focus was on trainee engagement with home-based simulation. However, an unanticipated finding was that trainer engagement with the programme was lacking. Trainers seemed either unaware of the programme, not confident in their role in delivering training and/or distant from the training process overall, which may be a deficiency of the programme organisation or of individual trainers’ capacity.

While these barriers are all reasonable, a less explicit barrier to trainer engagement (or lack of) may stem from the divergent positions of trainees and trainers in respect to trainees taking responsibility for their training. While awareness of the programme could be increased through local meetings and conferences, news bulletins and publications, and trainers could be supported to develop feedback competencies through the provision of ‘train the trainer’ courses, addressing a gap in expectations, or attitudes, is more challenging, as these are often ingrained in practice and the surgical culture [27]. Surgical systems factors may also influence trainer engagement. Good training takes time, but consultants (“attending surgeons”) have multiple competing commitments requiring their attention [28]. The desire to engage with simulation may exist, but the time to do so may not.

A major strength of this study is that viewpoints have been gathered from multiple stakeholders from various institutions throughout the UK and Ireland, hopefully making the results broadly applicable. Indeed, we urge those working in other contexts to consider these findings, as cross context/country/training system comparisons will illuminate the extent of this issue. Further, this study was exploratory and iterative, with themes identified from earlier interviews investigated in subsequent focus groups.

A potential limitation of this study was the ‘insider’ status of the lead researcher and interviewer, i.e. she was a surgical trainee. Her own preconceptions and biases may have influenced data collection and analysis. In an attempt to engage with reflexivity, the lead researcher aimed to gather and analyse data with her ‘eyes open’ and to avoid making assumptions [29]. Further, data analysis was overseen by a diverse, multidisciplinary team (surgeons, a psychologist, a general practitioner), and data interpretation was done on a group basis to ensure multiple viewpoints, leading to a process of discussion, sense making and consensus building.

Focus groups can have limitations. Certain personalities may dominate the discussion and the presence of some group members may discourage individuals from speaking openly and honestly. In an attempt to mitigate this, each focus group consisted of one type of stakeholder population (e.g. consultant trainer or naïve trainee). Though focus groups may not gather the depth of information associated with individual semi-structured interviews (SSI), they were a pragmatic way in which to gather views of multiple groups of individuals [30]. They are also an established method of exploring ‘the gap between what people say and what people do’. Finally, individuals may feel uncomfortable discussing these apparent shortcomings in a SSI setting, but a focus group deflects attention through the presence of other participants [30].

Our findings have implications for policy in surgical training, and change is already on the horizon. Promoting a shift in surgical culture away from a ‘tick box’ approach towards a focus on the development of excellence may help to re-frame trainee priorities in relation to their personal development. The Improving Surgical Training (IST) pilot, commenced in 2018 in several “deaneries” in the UK (including Scotland), may help in addressing this change in part through the introduction of modified assessment structures and run-through training [31, 32]. The IST programme may also help to develop tools for trainers to make them more effective and accountable in their training role as well as better acknowledging their contributions to training [31].

In conclusion, trainees are strategic in their approach to training and prioritise activities associated with career progression rather than tasks which help to support skill development. Unfortunately, surgical training can perpetuate this problem and trainees see themselves as simply ‘playing the game’. In order to promote a shift away from this ‘tick box’ culture we need to change the rules of the game.

## Appendix

### A discussion guide: familiar trainees

#### Facilitator’s welcome, introduction and instructions to participants

Facilitator: *Welcome and thank you for volunteering to take part in this discussion. You have been asked to participate as your point of view is important. I realise you are very busy and I appreciate your time*.

*The purpose of this interview is to discuss your views about the Incentivised Laparoscopy training programme* (or equivalent regional programme).

*If at any time you change your mind and wish to leave the focus group that is not a problem. Anything you have said up until that point will be deleted*.

#### Anonymity/rules

Facilitator: *Despite this interview being audiotaped, I would like to assure you that the discussion will be anonymous and anything that anyone says in the group should be treated as confidential. After the discussion, the audiotapes will be transcribed. During the transcription process you will be given a pseudonym so you can’t be identified*.

*They are no right or wrong answers – the idea of this group is to understand the range of perspectives*.

*Due to the limited time available, I may have to re-direct our discussion with a few questions*.

#### Setting the scene

Facilitator: *Do you have any questions?*

Actions: Consent forms—handout and complete preliminary questionnaire.

Facilitator: *For the purpose of transcription, can you all introduce yourselves please? If you can tell me your name and where you are working*.

#### Discussion outline

*Are you familiar with the term deliberate practice in the context of simulation based education?*

If yes, what do you understand it to mean?

If no, interviewer to explain. Deliberate practice is:

the repeated act of intended cognitive or psychomotor skills with the goal of improving overall performance. Feedback is a key element of deliberate practice.

*How is deliberate practice relevant to surgeons in training, or indeed other craft specialties?*

*How do the principles of deliberate practice align with the delivery of the incentivised laparoscopy training programme (ILPS) or equivalent programme?*

*What did you like about the ILPS (or equivalent) programme?*

*What were the problems with the ILPS (or equivalent) programme?*

*If you were designing a future laparoscopic simulation programme how would you improve it?*
